# Reply: “Letter to the editor Re: Diaz M., et al. *Nutrients* 2018, *10*, 1481”

**DOI:** 10.3390/nu11020476

**Published:** 2019-02-24

**Authors:** María Díaz, Lucía Guadamuro, Irene Espinosa-Martos, Leonardo Mancabelli, Santiago Jiménez, Cristina Molinos-Norniella, David Pérez-Solis, Christian Milani, Juan Miguel Rodríguez, Marco Ventura, Carlos Bousoño, Miguel Gueimonde, Abelardo Margolles, Juan José Díaz, Susana Delgado

**Affiliations:** 1 Department of Microbiology and Biochemistry of Dairy Products, Instituto de Productos Lácteos de Asturias (IPLA)-Consejo Superior de Investigaciones Científicas (CSIC), 33300 Villaviciosa-Asturias, Spain; Maria.Diaz@quadram.ac.uk (M.D.); luciagg@ipla.csic.es (L.G.); mgueimonde@ipla.csic.es (M.G.); amargolles@ipla.csic.es (A.M.); 2 Department of Nutrition and Food Science, Universidad Complutense de Madrid (UCM), 28040 Madrid, Spain; irene.espinosa@probisearch.com (I.E.-M.); jmrodrig@vet.ucm.es (J.M.R.); 3 Laboratory of Probiogenomics, Department of Chemistry, Life Sciences and Environmental Sustainability, University of Parma, 43121 Parma, Italy; leonardo.mancabelli@genprobio.com (L.M.); christian.milani@unipr.it (C.M.); marco.ventura@unipr.it (M.V.); 4 Paediatric Gastroenterology and Nutrition Section, Hospital Universitario Central de Asturias (HUCA), 33011 Oviedo-Asturias, Spain; principevegeta@hotmail.com (S.J.); ringerbou@yahoo.es (C.B.); 5 Paediatrics, Hospital Universitario de Cabueñes, 33394 Gijón-Asturias, Spain; cristinamolinos@gmail.com; 6 Paediatrics, Hospital Universitario San Agustín, 33401 Avilés-Asturias, Spain; doctorin@gmail.com

The objective of this letter of reply is to provide answers to the doubts and critical issues that Martín Martinez and López Liñan [1] raised in their letter to the editor with respect to the work published last year in *Nutrients* in the field of microbiota, diet and non-IgE cow's milk protein allergy (NIM-CMPA).

As authors of this publication, we understand and welcome the controversial issues that unavoidably surround any novel research results with implications for the dietary management of cow's milk protein allergy (CMPA).

First, we would like to point out that in the latest published recommendations on CMPA, the allergologic and nutritional safety of hydrolyzed rice protein formulas (HRF) according to DRACMA (Diagnosis and Rationale for Action against Cow’s Milk Allergy) guidelines are clearly stated. However, according to this review it is advisable that debate is ongoing about the best substitute for infants with CMPA, and it concludes that “in the substitute choice, clinicians should be aware of recent studies that can modify the interpretation of the current recommendations” [2]. The letter to the editor of Martín Martinez and López Liñan argues in favor of the use of HRF, which is a reasonable position but which must be open to debate in the light of new data. As mentioned in our publication, “the data found in this pilot study need to be confirmed in a larger population” [3], thus, we cannot disagree with Martín Martinez and López Liñan that the main limitation of our study is the small number of patients included. Nonetheless, this limitation is already mentioned in our paper. Further research studies are of course needed and we remain open-minded, but a lack of consensus does not represent a lack of evidence. 

With respect to the first questionable aspect of our study; as stated in the publication [3], our work is a prospective cohort study, so infants were not randomly assigned to each formula, however, we really do not think this constitutes a limitation of the study since it was an observational, not an interventional study. In this case, randomization was not intended in the study, which focused on the description of what was happening (microbiota and biochemical parameters of fecal samples) at the intestinal level in non-IgE mediated cow's milk protein allergy (NIM-CMPA) infants as compared with healthy controls, for the first time as far as we know. Infants with proven NIM-CMPA determined by positive standardized oral challenge (SOC) and negative IgE tests to cow´s milk proteins (CMP) were included in our study when they were on a cow´s milk elimination diet for at least 6 months. The substitution formula was decided by the attending physician, and therefore, the number of infants on each formula simply reflects the formula selection by pediatric gastroenterologists in Spain. In a recently published survey, extensively hydrolyzed whey or casein formulas were those of choice, followed by amino acid-based formulas, HRF and soy formulas [4]. Furthermore, formula selection was not the primary variable outcome of our study. 

Secondly, Martín Martinez and López Liñan [1] mentioned that the authors did not provide enough information about the diet of the infants before inclusion, and because the studied infant ages ranges between 13 and 23 months, we should provide information about the type of diet that infants received from diagnosis until the beginning of the study. Probably we did not make these points clear enough in our manuscript. The age of the infants was recorded at the time of fecal sampling, after a period of at least 6 months using therapeutic hypoallergenic formulas as the main dietary food, and matching with the age of the control group (median 18 months versus 17 months in the NIM-CMPA group). That means that at the time of diagnosis, the NIM-CMPA infants were at least 6 months younger than at sampling. In their letter [1], Martín Martinez and López Liñan argue that “another important difference between controls and infants with NIM-CMPA is that the latter followed a diet free of dairy products for 6 months. Indeed, this can be a factor modifying the microbiota”. We believe that their interpretation overlooks important points of our study. Control infants are healthy infants that consume milk and dairy without restriction, and of course, infants with NIM-CMPA are on a restricted diet for at least 6 months. Hence, we did not conclude that differences in microbiota are only due to the allergic condition of the infants, and of course, we think that a milk restricted diet is at least partially responsible for the observed differences.

It is also suggested that another missing piece of information in our study is related to the type of formula, which has a strong impact on microbiota. It is true that we have not provided detailed information on the type of hydrolyzed formula used in our study (trademarks) apart from extensively hydrolyzed formula (EHF), soy protein-based formulas, and HRF, and maybe this has contributed to some of the confusion. Nevertheless, significant differences in microbiota composition were found between those infants fed with vegetable protein-based formulas and those consuming EHF, but not among the last group. Additionally, regarding the argument focusing on lactose, it is important to underline that in infants with IgE mediated CMPA, formulas containing lactose are sometimes used. However, in NIM-CMPA cases, these formulas are usually avoided. 

Surprisingly, another point that is criticized is the analysis of the microbiota. Our microbial sequences are deposited in a public database and openly available [3], allowing further analyses by other authors. We found that those infants who do not become tolerant in the study (fed HRF) present significant differences and less abundance of Coriobacteriaceae and Bifidobacteriaceae members than those who become tolerant. It is also noticeable in our study, that infants fed vegetal protein formulas, both rice and soy, have significantly less Coriobacteriaceae than those fed EHF formulas. The details on the levels are displayed on page 5: “Coriobacteriaceae were significantly diminished in NIM-CMPA infants consuming vegetable protein-based formulas (n = 5), both rice and soy, (mean of 0.64% of total assigned reads, range 0.02–2.38%) compared to those infants fed with EHF (mean of 4.45%, range 0.08–13.44%)”, and in Table 1 (Coriobacteriaceae mean abundance in infants consuming HRF = 0.26%) [3]. A potential explanation for these observations is mentioned in the discussion section of our article in relation to cross-feeding mechanisms among lactate microbial users and butyrate producers, although it is true that the exact role of the Coriobacteriaceae in the infant gut microbiota is still unknown. Moreover, we do not have a clear response to the authors of this letter as to why these Coriobacteriaceae members are enriched in those infants fed with EHF, as compared also with healthy controls infants, although, a positive significant correlation was found between this family and butyrate levels [3]. In addition, a statistical association was detected between this important microbial metabolite and the excretion of the mediator TGF-β, implicated in tolerance, so there is a potential relationship among all these factors.

Regarding the comment on the use of vegetable formulas and the possible impairment of tolerance acquisition, Martín Martinez and López Liñan [1] indicate that “this conclusion cannot be drawn from the results of the study from Diaz et al”. However, although these issues are discussed within the discussion section, our manuscript does not establish such a conclusion. Actually, our conclusions are that type of formula can determine microbiota composition, and that this, through the production of microbial metabolites and modulation of immune mediators, may influence tolerance acquisition. Of course, in the discussion we are allowed to comment on possible explanations for our results, based on current knowledge such as exposure to immunomodulatory peptides.

We hope this letter will help to clarify the points raised by Martín Martinez and López Liñan and appreciate the interest of these authors and the opportunity to engage in a dialogue around NIM-CMPA tolerance, diet and microbiota. 
